# LINC_00355 promotes gastric cancer progression by upregulating PHF19 expression through sponging miR-15a-5p

**DOI:** 10.1186/s12885-021-08227-3

**Published:** 2021-06-02

**Authors:** Jishui Zhang, Wenhao Lv, Yagang Liu, Weihua Fu, Baosheng Chen, Qiutong Ma, Xin Gao

**Affiliations:** 1 Department of Gastrointestinal Surgery, Cangzhou Central Hospital, No. 16 Xinhua West Road, Cangzhou, 061001 Hebei China; 2 Department of General Surgery, Tianjin Medical University General Hospital, Tianjin, 300052 China; 3 Department of Radiotherapy, Cangzhou Central Hospital, Cangzhou, 061001 Hebei China

**Keywords:** Gastric cancer, LINC_00355, miR-15a-5p, PHD finger protein 19

## Abstract

**Background:**

Long non-coding RNAs exert vital roles in several types of cancer. The objective of this study was to explore the role of LINC_00355 in gastric cancer (GC) progression and its potential mechanism.

**Methods:**

The expression levels of LINC_00355 in GC tissues and cells were detected by quantitative real-time PCR, followed by assessing the effects of LINC_00355 knockdown or overexpression on cell properties. Dual-luciferase reporter assay was utilized to identify the relationship between LINC_00355 and microRNA (miR)-15a-5p and miR-15a-5p and PHD finger protein 19 (PHF19), followed by the rescue experiments.

**Results:**

The results showed that LINC_00355 was highly expressed in GC tissues and cells compared with the corresponding control. LINC_00355 knockdown decreased the viability, migration, and invasion and increased the accumulation of GC cells in G1 phase and apoptosis. Meanwhile, LINC_00355 downregulation markedly increased cleaved caspase 3 and cleaved poly (ADP-ribose) polymerase protein levels, whereas decreased cyclin D1, cyclin E, matrix metalloproteinase (MMP) 9, MMP2, and N-cadherin protein levels in GC cells. However, LINC_00355 overexpression had the opposite effects. It was verified that LINC_00355 upregulated the expression of PHF19 through sponging miR-15a-5p. Furthermore, PHF19 overexpression reversed the effect of LINC_00355 knockdown on GC cell properties, including cell viability, migration, invasion, and apoptosis.

**Conclusions:**

Collectively, these results suggest that LINC_00355 promotes GC progression by up-regulating PHF19 through sponging miR-15a-5p. Our findings may provide an important clinical basis for reversing the malignant phenotype of GC.

**Supplementary Information:**

The online version contains supplementary material available at 10.1186/s12885-021-08227-3.

## Background

Gastric cancer (GC) is one of the most common cancers in the world and the third leading cause of cancer death [1]. According to statistics in 2018, there were 1,033,000 newly diagnosed GC cases and 783,000 deaths worldwide, indicating that GC has become a serious threat to human health [2]. In recent years, with the development of colonoscopy and surgical techniques, the 5-year mortality rate of early GC has been significantly reduced. However, for advanced GC, the 5-year mortality rate was still high [3]. Therefore, exploring the mechanism of GC development and potential therapeutic targets may provide a new idea for the treatment of GC.

Long noncoding RNA (lncRNA) is a non-coding RNA with a length of more than 200 nt. LncRNA can act as a tumor inducer or suppressor in the development of cancers [4, 5]. It has been found that LINC_00355 was highly expressed in bladder transitional cell carcinoma [6]. LINC_00355 was involved in the regulation of competitive endogenous RNA (ceRNA) networks and was differentially expressed in multiple pathological stages of colorectal cancer [7]. Previous findings indicated a vital role of LINC_00355 in cancer development. However, whether LINC_00355 plays a role in the development of GC remains unknown.

There is increasing evidence that lncRNA plays the role of ceRNA. LncRNA can regulate the expression of target genes through competitively binding of microRNA (miRNA) [8, 9]. The ceRNA hypothesis provides a new idea for the identification of lncRNA functions [10]. Wei et al. [11] found that LINC_00355 was highly expressed in lung adenocarcinoma, and LINC_00355 promoted the proliferation and colony formation of lung adenocarcinoma cell and inhibited cell cycle arrest and apoptosis through competitively binding to miR-195. Lu et al. [12] showed that LINC_00355 served as a miR-195 sponge to enhance viability, invasion, migration, and inhibit apoptosis of head and neck squamous cell carcinoma (HNSCC) cells by increasing homeoboxA10 expression. In present study, we focused on the role of lncRNA LINC_00355 in GC. We hypothesized that LINC_00355 might be involved in the development of GC via regulating miRNA/mRNA axis.

## Methods

### Sample and clinical data collection

GC tissues and adjacent tissues (*n* = 30) were collected from the Cangzhou Central Hospital. The patients did not receive any surgery, chemotherapy, radiation, or other anti-cancer treatment.

### Cell culture and transfection

The GC cells lines (AGS, SNU-1, MKN45, HGC-27) were purchased from Procell Life Science&Technology Co.,Ltd. (Wuhan, China), Fuheng Biotechnology Co., Ltd. (Shanghai, China), and Zhong Qiao Xin Zhou Biotechnology Co., Ltd. (Shanghai, China). HGC-27 cells were cultured in Roswell Park Memorial Institute (RPMI)-1640 medium (Hyclone, South Logan, UT, USA) supplemented with 20% fetal bovine serum (FBS, Hyclone). MKN45 cells were cultured in RPMI-1640 medium (Hyclone) supplemented with 10% FBS (Hyclone). These cells were incubated in an incubator at 37 °C with 5% CO_2_.

HGC-27 and MKN45 cells were seeded in 6-well plates with a density of 4 × 10^5^ cells/well and then cultured in an incubator at 37 °C with 5% CO_2_. After 24 h incubation, cells were transfected with siRNA LINC_00355 (si-LINC_00355), pcDNA3.1-PHF19 plasmid or its negative control (si-NC/vector) for 24 h. For the co-transfection, HGC-27 and MKN45 cells were co-transfected with si-LINC_00355 and pcDNA3.1-PHD finger protein 19 (PHF19) plasmid for 24 h or 48 h. The transfections were mediated by Lipofectamine™ 2000 (Invitrogen, Carlsbad, CA, USA).

### RNA isolation and quantitative real-time PCR (RT-qPCR) analysis

Total RNA were isolated from tissues and cells by using total RNA extraction kit (BioTeke, Beijing, China) according to the manufacturer’s instructions. RNA was then reverse-transcribed to complementary DNA (cDNA) by using a reverse transcriptase kit (Takara, Dalian, China). RT-qPCR was conducted using cDNA, primers, SYBR Green, and Taq HS Perfect Mix. Primers are synthesized by GenScript Biotechnology Co., Ltd. (Nanjing, China). The β-actin was used as a loading control for LINC_00355 and PHF19. U6 served as a loading control for miR-15a-5p. The relative expression levels of genes were calculated by using 2^−ΔΔct^ analysis.

### Cell viability assay

Cell viability was measured by Cell Counting Kit-8 assay. Cells were seeded in 96-well plates with a density of 3 × 10^3^ cells/well. After transfection, cells were placed in an incubator for 0, 6, 24, 48, and 72 h, respectively, at 37 °C with 5% CO_2_. Subsequently, CCK-8 (10 μl) was added to each well and incubated at 37 °C in 5% CO_2_ for 1 h. For each well, the optical density value was measured at a wavelength of 450 nm.

### Cell apoptosis analysis

Cells were placed in 6-well plates with a density of 4 × 10^5^ cells/well. After transfection, cells were harvested and treated with AnnexinV-FITC (5 μl) and propidium iodide (PI, 10 μl). After incubation at room temperature in the dark for 15 min, cells were resuspended in 1 × binding buffer. The percentage of apoptotic cells was calculated by using flow cytometry (Aceabio, San Diego, CA, USA).

### Cell cycle analysis

Cells were seeded into 6-well plates at a density of 4 × 10^5^/well. After transfection, cells were harvested and fixed in ethanol (70%) at 4 °C for 12 h. Subsequently, the cells were collected by centrifugation and resuspended in staining buffer and incubated with PI staining solution (25 μl) and RNase A (10 μl) in the dark for 30 min. Flow cytometry (Aceabio) was used to detect cell cycle.

### Western blot analysis

Total protein was extracted from cells and separated with SDS polyacrylamide gel electrophoresis. The proteins were then transferred onto the polyvinylidene difluoride (PVDF) membranes. The membranes were incubated with anti-cyclin D1 (1:1000, Cell Signaling Technology (CST), Danvers, MA, USA), anti-cyclin E (1:500, Affinity Biosciences, Cincinnati, OH, USA), anti-pro/cleaved caspase 3 (1:500, CST), anti-pro/cleaved poly (ADP-ribose) polymerase (PARP) (1:1000, CST), anti-matrix metalloproteinase (MMP) 2 (1:1000, CST), anti-MMP9 (1:1000, CST), anti-N-cadherin (1:500, CST), anti-PHF19 (1:500, Affinity Biosciences), and anti-β-actin (1:2000, Proteintech, Wuhan, China) at 4 °C overnight. After washing three times (5 min/time) with TBST, the membranes were incubated with horseradish peroxidase-conjugated goat anti-rabbit/mouse IgG (1:10000, Proteintech) for 2 h. After washing three times (5 min/time) with TBST, the membranes were visualized by treated with electrochemiluminescence luminescent solution. β-actin served as a loading control for protein expression.

### Cell migration assay

Cell migration was assessed by wound-healing assay. The cells of each group were cultured to the density of fusion state. Before the experiment, the medium was changed to serum-free medium and treated with mitomycin C (1 μg/ml) for 1 h. The cells were scratched with 200 μl pipette tip and the surface of the cells was washed once with serum-free medium to remove cell debris. Cells were then incubated in an incubator at 37 °C with 5% CO_2_ for 0 h and 24 h, respectively, and then photographed, and the location of the cells was recorded.

### Cell invasion assay

Cell invasion was assessed by transwell assay. Transwell chamber coated with Matrigel gel was placed into a 24-well plate. Culture medium (800 μl) containing 10% FBS was added to the lower chamber and cell suspension (200 μl) was added to the upper chamber (2 × 10^4^ cells/well). The 24-well plates were cultured in a cell incubator at 37 °C with 5% CO_2_. Subsequently, after fixed in 4% paraformaldehyde at room temperature for 25 min, the cells were stained with 0.4% crystal violet staining solution for 5 min and rinsed with distilled water. Cells migrating to the sublayer of the microporous membrane were counted under an inverted microscope (Olympus, Tokyo, Japan).

### Dual-luciferase reporter assay

The binding sites of LINC_00355 and miR-15a-5p were predicted by bioinformatics analysis. (LncBase v.2, http://carolina.imis.athena-innovation.gr/diana_tools/web/index.php?r=lncbasev2/index-predicted). The binding sites of miR-15a-5p and PHF19 were predicted by TargetScanHuman 7.2 (http://www.targetscan.org/vert_72/). The wild type (wt) and mutant (mut) fragments of LINC_00355 were cloned into pGLO vector (GenScript, Nanjing, China) to construct LINC_00355-wt and LINC_00355-mut plasmids. The 3′-untranslated region (3′-UTR) sequences of PHF19 containing predicated or mutated miR-15a-5p binding sites were used to construct PHF19 3′-UTR-wt and PHF19 3′-UTR-mut plasmids. Cells were co-transfected with miR-15a-5p mimic (12.5 or 25 pmol per well) or its NC (12.5 or 25 pmol per well) and corresponding reporter plasmids (0.5 μg per well) using Lipofectamine™ 2000 (Invitrogen). At 48 h after transfection, luciferase assay kit (KeyGEN, Jiangsu, China) was used to detect luciferase activity.

### Statistical analysis

All data analysis was conducted using GraphPad version 8.0. Comparisons between groups were estimated by the paired t-test, one-way ANOVA or two-way ANOVA. *P* < 0.05 was considered statistically significant.

## Results

### LINC_00355 was highly expressed in GC tissues and cell lines

**Fig. 1:**
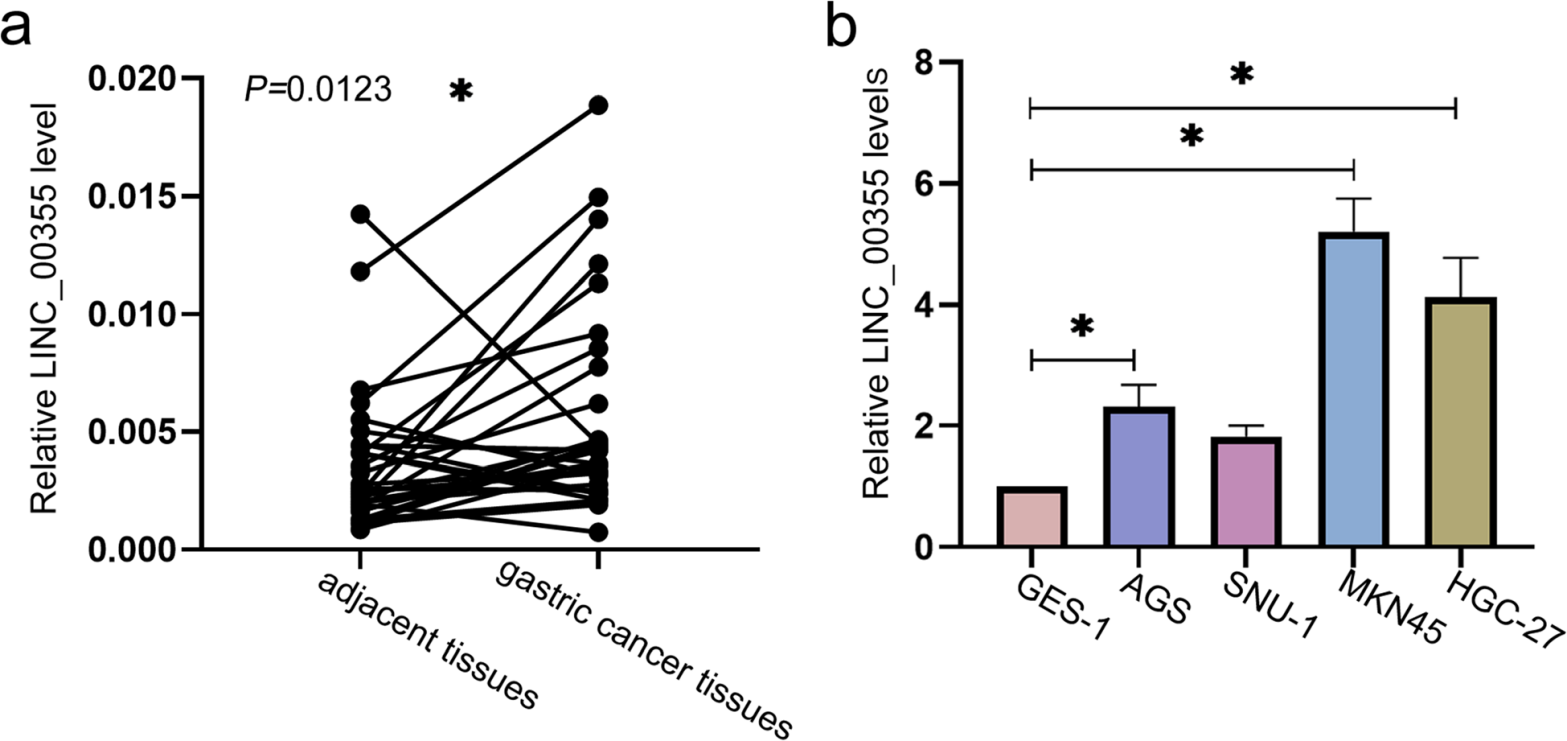
LINC_00355 was highly expressed in GC tissues and cell lines. **a** The expression levels of LINC_00355 in GC tissues and adjacent tissues (*n* = 30) detected by quantitative real-time PCR. *P* < 0.05 compared with adjacent tissues. **b** Relative LINC_00355 expression levels in GC cells lines (AGS, SNU-1, MKN45, HGC-27) and normal gastric cells (GES-1). All values were expressed as mean ± standard deviation. *n* = 3. *P < 0.05 compared with GES-1. GC, gastric cancer

We first explored the expression level of LINC_00355 in GC tissues and cells by RT-qPCR. As shown in Fig. 1a, the expression of LINC_00355 in 30 pairs of GC tissues was significantly higher than that of adjacent tissues (*P* = 0.0123). Meanwhile, the LINC_00355 expression levels in GC cells lines (AGS, MKN45, and HGC-27) were significantly increased as compared with normal gastric cells (GES-1) (Fig. 1b). Among them, MKN45 and HGC-27 cell lines with relatively high expression levels of LINC_00355 were used for subsequent experiments. Overall, the results indicated that LINC_00355 may be positively associated with the development of GC.

### LINC_00355 silencing inhibited the proliferation and promoted apoptosis in GC cells

**Fig. 2:**
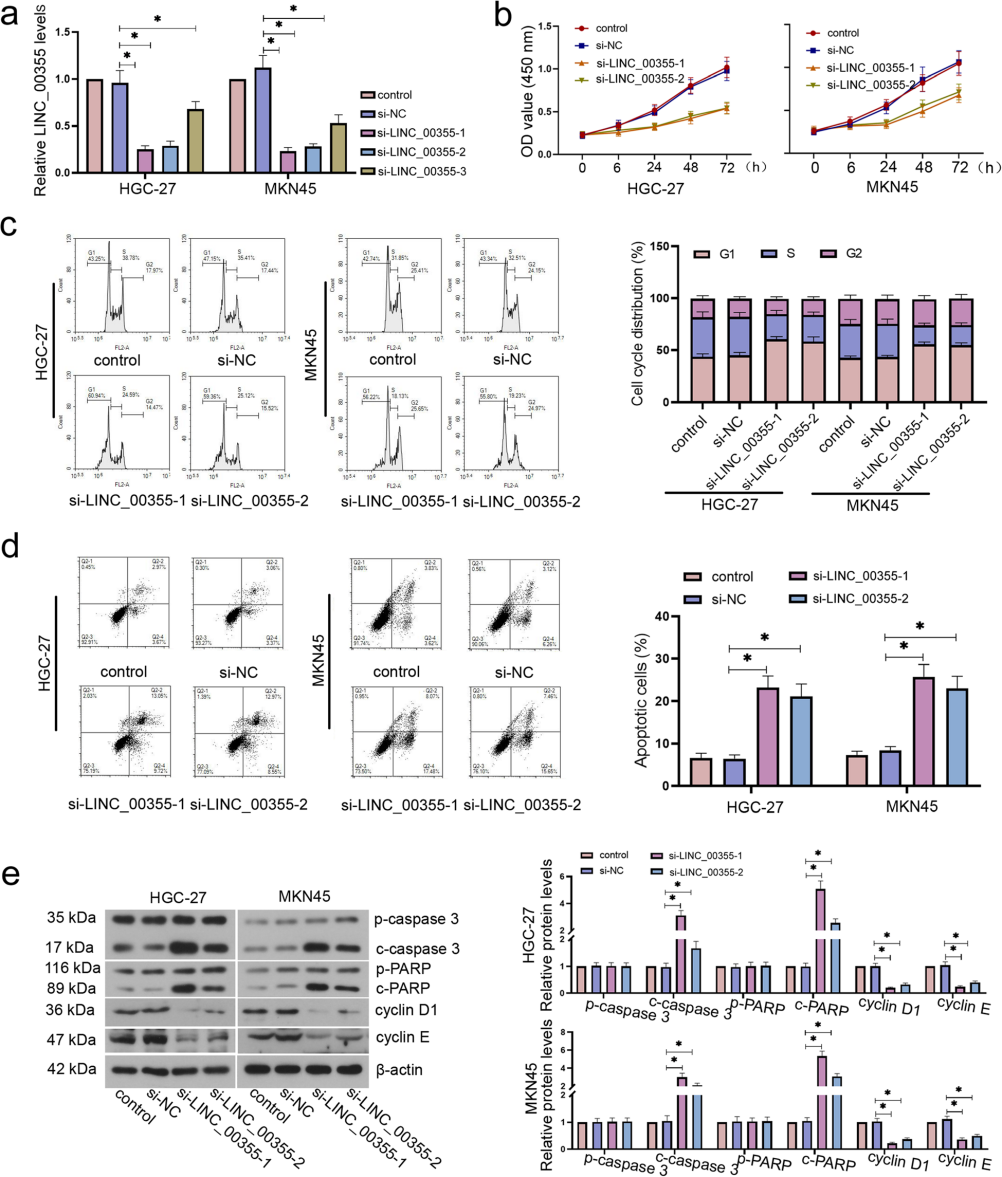
LINC_00355 silencing inhibited the proliferation and promoted apoptosis in GC cells. After HGC-27 and MKN45 cells were transfected with si-LINC_00355–1 and − 2 for 24 h, relative LINC_00355 expression levels in HGC-27 and MKN45 cells were detected by quantitative real-time PCR (**a**); The cell viability were measured by Cell Counting Kit-8 assay (**b**); Cell cycle distribution and apoptosis were measured by flow cytometer (**c** and **d**); Relative pro/cleaved caspase 3, pro/cleaved PARP, cyclin D1, and cyclin E protein levels were assessed using western blot analysis (**e**). All values were expressed as mean ± standard deviation. n = 3. *P < 0.05 compared with si-NC group. GC, gastric cancer; PARP, poly (ADP-ribose) polymerase; NC, negative control

To investigate the role of LINC_00355 in GC cell proliferation and apoptosis, we knocked down the expression of LINC_00355 by transfecting siRNA. The results showed that si-LINC_00355 transfection significantly decreased LINC_00355 expression compared with control group in HGC-27 and MKN45 cells (Fig. 2a). The results of CCK-8 detection showed that LINC_00355 silencing observably inhibited the viability in GC cells, whereas LINC_00355 overexpression increased the viability of GC cells (Fig. 2b and SF. 1c). The cell cycle distribution and apoptosis rate was detected by flow cytometry, and the results showed that LINC_00355 knockdown led to the accumulation of GC cells in the G1 phase and induced apoptosis (Fig. 2c and d). Meanwhile, the protein expression levels of apoptosis-related factors cleaved caspase 3 and cleaved PARP was markedly increased by LINC_00355 downregulation (Fig. 2e). The si-LINC_00355 transfection significantly reduced cyclin D1 and cyclin E protein levels in comparison with the si-NC group (Fig. 2e). Conversely, the protein level of cyclin D1 in GC cells was significantly increased by LINC_00355 overexpression (SF. 1f). Overall, the results indicated that LINC_00355 can promote cell viability and inhibit apoptosis during GC progression.

### LINC_00355 silencing suppressed the migration and invasion in GC cells

**Fig. 3:**
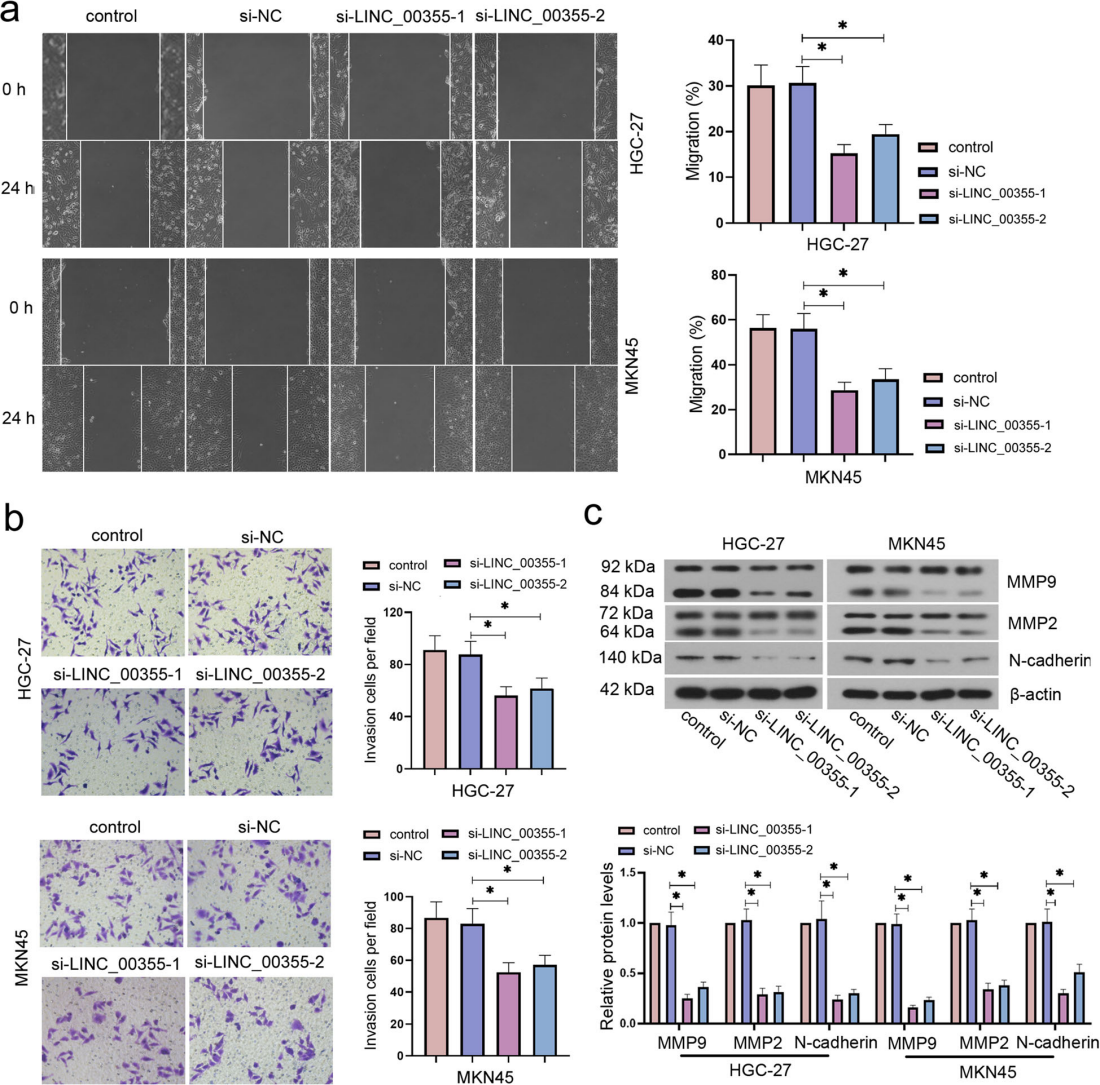
LINC_00355 silencing suppressed the migration and invasion in GC cells. After HGC-27 and MKN45 cells were transfected with si-LINC_00355–1 and − 2 for 24 h, cell migration was detected by wound-healing assay (**a**); cell invasion was measured by Transwell assay (**b**); Relatively matrix metalloproteinase (MMP) 9, MMP2, and N-cadherin protein levels were measured by western blot analysis (**c**). All values were expressed as mean ± standard deviation. n = 3. *P < 0.05 compared with si-NC group. GC, gastric cancer; NC, negative control

We further explored the effects of LINC_00355 knockdown on migration and invasion of GC cells. As presented in Fig. 3a, the migration and invasion ability of GC cells with LINC_00355 knockdown was lower than that in the si-NC group, whereas LINC_00355 upregulation showed the opposite effects (Fig. 3a and b and SF. 1d and e). Additionally, the protein expression levels of MMP9, MMP2, and N-cadherin were markedly decreased in si-LINC_00355–1 and si-LINC_00355–2 groups in comparison with the si-NC group (Fig. 3c). However, the MMP2 and MMP9 protein levels in LINC_00355-overexpressed cells were markedly increased (SF. 1f). Thus, these results demonstrate that LINC_00355 is positively correlated with the migration and invasion of GC cells.

### LINC_00355 up-regulated PHF19 expression through sponging miR-15a-5p in GC cells

**Fig. 4:**
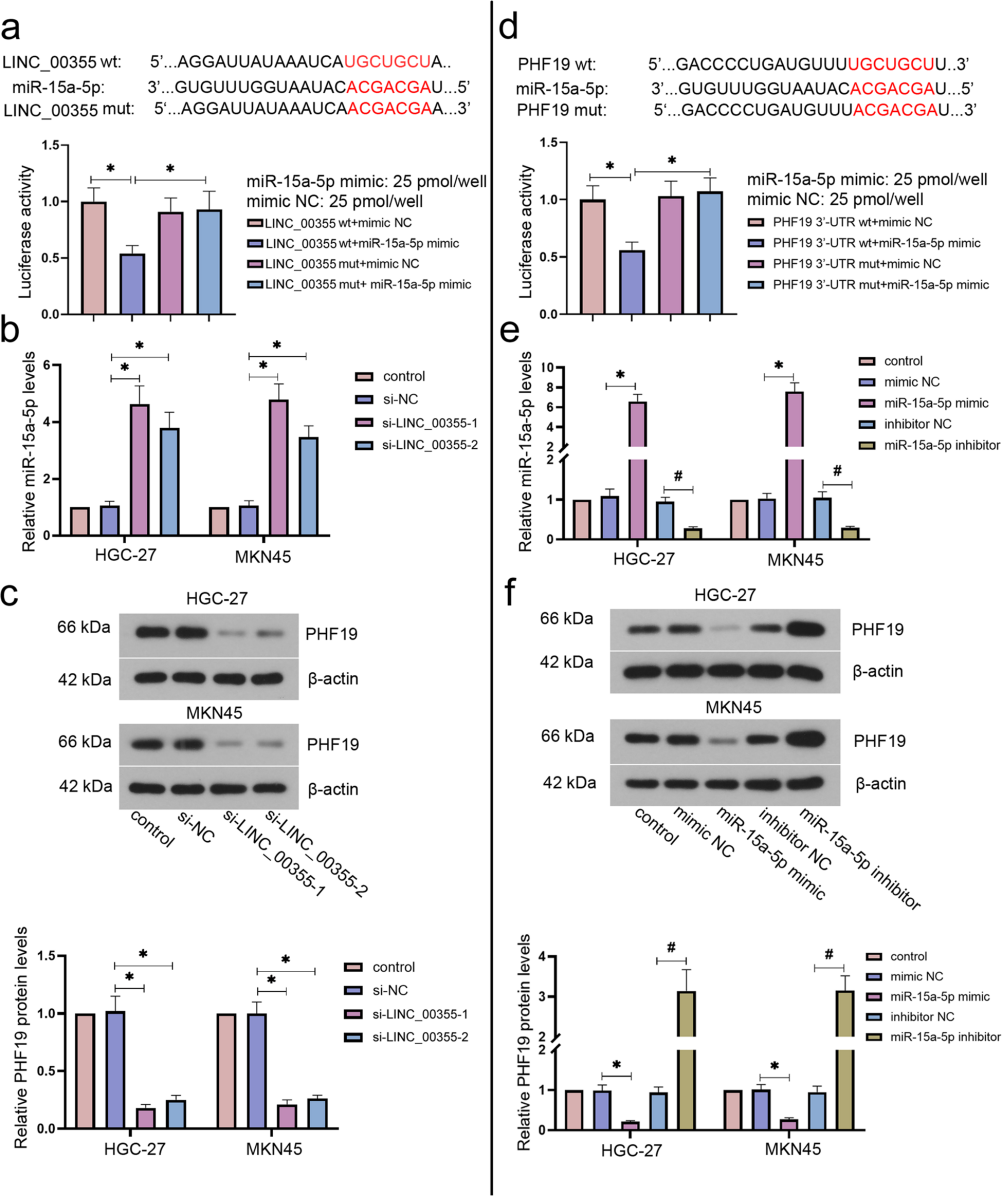
LINC_00355 upregulated PHF19 expression through sponging miR-15a-5p in GC cells. **a** Relative luciferase activity in 293 T cells co-transfected with LINC_00355 reporter plasmid and the candidate miRNA. *P < 0.05 compared with LINC_00355 wt + miR-15a-5p mimic group (**b**) Relative miR-15a-5p expression levels in HGC-27 and MKN45 cells transfected with si-LINC_00355–1 and − 2. *P < 0.05 compared with si-NC group. **c** Relative PHF19 protein expression levels in HGC-27 and MKN45 cells transfected with si-LINC_00355–1 and − 2. *P < 0.05 compared with si-NC group. **d** Relative luciferase activity in 293 T cells co-transfected with PHF19 reporter plasmid and the candidate miRNA. *P < 0.05 compared with PHF19 3′-UTR wt + miR-15a-5p mimic group. **e** The expression levels of miR-15a-5p in HGC-27 and MKN45 cells transfected with miR-15a-5p mimic, inhibitor, or its corresponding negative control. *P < 0.05 compared with mimic NC group and ^#^*P* < 0.05 compared with inhibitor NC group. **f** Relative PHF19 protein levels in HGC-27 and MKN45 cells transfected with miR-15a-5p mimic or inhibitor. *P < 0.05 compared with mimic NC group and ^#^*P* < 0.05 compared with inhibitor NC group. All values were expressed as mean ± standard deviation. n = 3. GC, gastric cancer; NC, negative control

We further explored the specific mechanism of LINC_00355 involved in the development of GC. Bioinformatics analysis predicted the binding sites between LINC_00355 and miR-15a-5p (Fig. 4a). Moreover, the results of dual-luciferase reporter assay indicated that the luciferase activity in LINC_00355 wt was significantly decreased by miR-15a-5p mimic in a dose-dependent manner and no significant changes was observed in other groups (Fig. 4a and SF. 2). The expression level of miR-15a-5p in GC cells was detected by RT-qPCR and showed that the depletion of LINC_00355 significantly increased miR-15a-5p expression levels in HGC-27 and MKN45 cells (Fig. 4b). On the contrary, the protein expression levels of PHF19 in HGC-27 and MKN45 cells were decreased by LINC_00355 downregulation (Fig. 4c). Bioinformatics analysis predicted the binding sites between miR-15a-5p and PHF19 (Fig. 4d). Luciferase assays indicated that the luciferase activities of PHF19 3′-UTR-wt were decreased by miR-15a-5p mimic dose-dependently (Fig. 4d and SF. 2). Results of RT-qPCR analysis showed that the expression level of miR-15a-5p was significantly increased by miR-15a-5p mimic and decreased by miR-15a-5p inhibitor in HGC-27 and MKN45 cells (Fig. 4e). However, the protein level of PHF19 was markedly decreased by miR-15a-5p overexpression and increased by miR-15a-5p knockdown (Fig. 4f). Collectively, the results indicate that LINC_00355 acts as a ceRNA to regulate PHF19 expression via competitively binding to miR-15a-5p.

### PHF19 overexpression reversed the effect of LINC_00355 knockdown on the development of GC

**Fig. 5:**
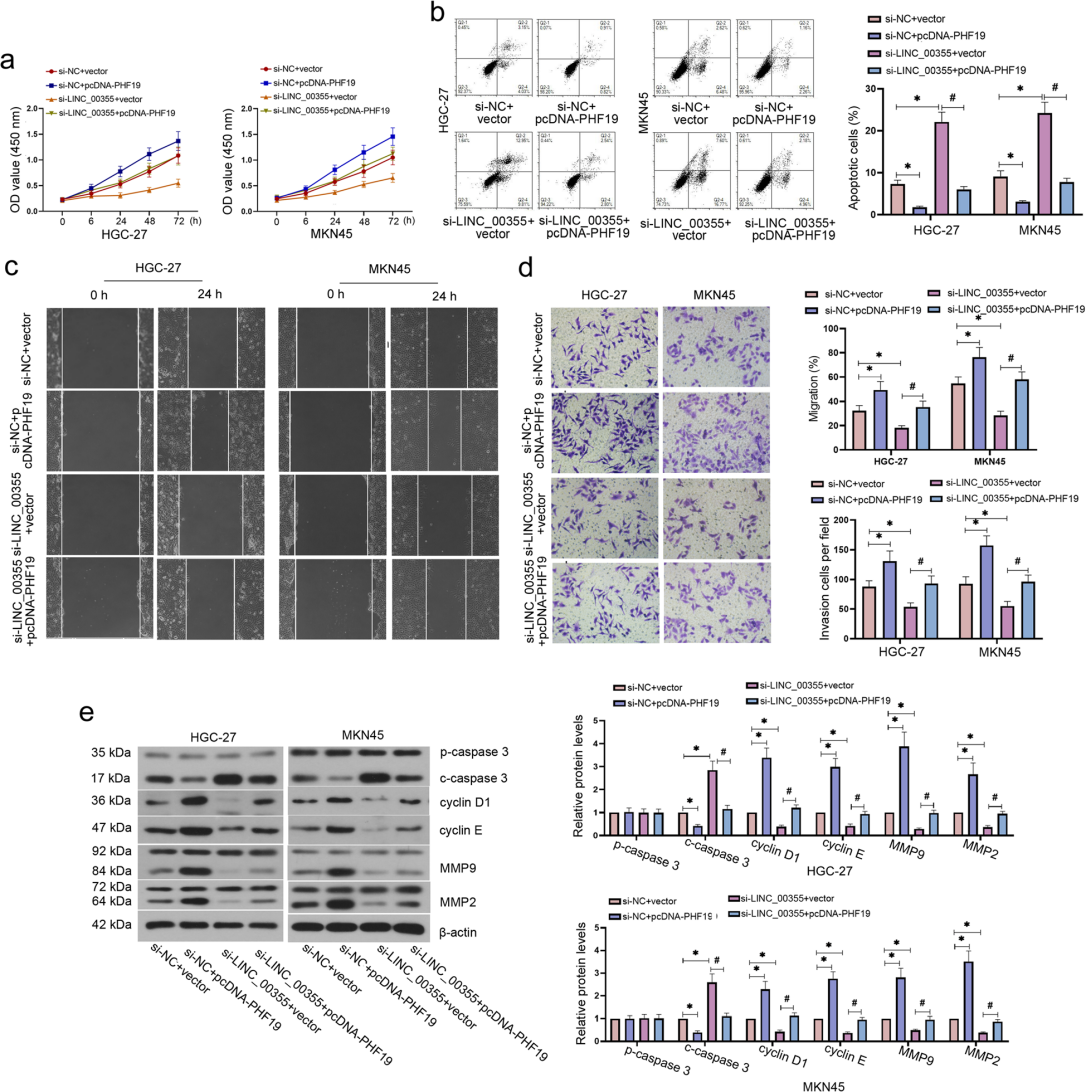
PHF19 overexpression reversed the effect of LINC_00355 knockdown on the development of GC. After HGC-27 and MKN45 cells were co-transfected with si-LINC_00355 and pcDNA3.1-PHF19 plasmid, cell viability, migration, invasion, and apoptosis were detected (**a**-**d**). The expression levels of pro/cleaved caspase 3, cyclin D1, cyclin E, matrix metalloproteinase (MMP) 9, and MMP2 were detected by western blot analysis (**e**). All values were expressed as mean ± standard deviation. *n* = 3. *P < 0.05 compared with si-NC + vector group and ^#^*P* < 0.05 compared with si-LINC_00355 + vector group. GC, gastric cancer; NC, negative control

We next analyzed the correlation between LINC_00355 and PHF19 expression via Pearson correlation analysis. The results showed that the expression of PHF19 in GC tissues (*n* = 30) was positively correlated with LINC_00355 (SF. 1 g). To verify whether LINC_00355 plays a role in GC progression through regulating PHF19 expression, HGC-27 and MKN45 cells were co-transfected with si-LINC_00355 and pcDNA3.1-PHF19 plasmid. As shown in SF. 1a, the transfection efficiency of pcDNA3.1-PHF19 plasmid was first verified in HGC-27 and MKN45 cells. PHF19 overexpression significantly increased viability, invasion, and migration and inhibited apoptosis in GC cells in comparison with the si-NC + vector group (Fig. 5a-d). Moreover, PHF19 overexpression reversed the effect of LINC_00355 knockdown on GC cell functions, including cell viability, migration, invasion, and apoptosis (Fig. 5a-d). Additionally, PHF19 upregulation markedly decreased the protein expression levels of cleaved caspase 3 and cleaved PARP, whereas significantly increased cyclin D1, cyclin E, MMP9, and MMP2 protein levels as compared with si-NC + vector group in HGC-27 and MKN45 cells (Fig. 5e). Furthermore, PHF19 upregulation inverted the effect of LINC_00355 silencing on the protein expression levels of cleaved caspase 3, cleaved PARP, cyclin D1, cyclin E, MMP9, and MMP2 (Fig. 5e). Overall, these results indicate that LINC_00355 may promote the development of GC by sponging miR-15a-5p to regulate PHF19 expression.

## Discussion

This study was the first to investigate the role of LINC_00355 in the development of GC and its potential mechanisms. We demonstrated that LINC_00355 was highly expressed in GC tissues and cells. LINC_00355 knockdown inhibited the proliferation, migration, and invasion and induced apoptosis of GC cells. Furthermore, LINC_00355 increased the expression of PHF19 by competitively inhibiting its binding to miR-15a-5p. Moreover, PHF19 was identified as a facilitator of GC development. Therefore, the present study demonstrated that LINC_00355 may promote the development of GC by up-regulating miR-15a-5p-mediated PHF19 expression.

Various studies have shown that lncRNA plays a vital role in regulating cell processes, including cell cycle, growth, and apoptosis [13, 14]. LncRNA changes at the transcriptional or post-transcriptional levels directly or indirectly lead to uncontrolled tumor growth [15, 16]. It was previously reported that LINC_00355 was associated with the clinical features of colorectal cancer and negatively correlates with survival rate [7]. Additionally, LINC_00355 was highly expressed in prostate cancer and associated with the survival rates of cancer patients [17]. Here, highly expressed LINC_00355 was also observed in GC tissues and cells, indicating that LINC_00355 may play a promoting role in the development of GC. Further studies indicated that downregulation of LINC_00355 suppressed the viability, invasion, migration, and promoted apoptosis of GC cells. LINC_00355 inhibition also improves the malignant phenotype of other cancers. For example, LINC_00355 knockdown inhibited the proliferation and invasion of bladder cancer cells [18]. The downregulation of LINC_00355 inhibited the viability, invasion, migration, and epithelial-mesenchymal transition and promoted apoptosis of cancer stem cells in HNSCC [12]. Collectively, LINC_00355 may participate in the development of GC by regulating the functions of GC cells, including proliferation, migration, invasion, and apoptosis.

More and more evidence supported the ceRNA hypothesis and showed that the ceRNA regulatory network was closely related to the development of GC. Bioinformatics analysis showed that there were binding sites between LINC_00355 and miR-15a-5p, which was verified by dual-luciferase reporter assay. Notably, a single lncRNA often plays a role in cancer by sponging multiple miRNAs. For instance, LINC_00355 promoted HNSCC progression through binding miR-195 [12]. In hepatocellular carcinoma (HCC), LINC_00355 acted as a ceRNA to sponge miR-6777-3p and further promoted HCC progression [19]. These findings indicate that LINC_00355 regulates cancer development in multiple pathways and highlights the important role of LINC_00355 in cancer progression. In present study, we demonstrate that LINC_00355 promotes GC progression through sponging miR-15a-5p, indicating that the LINC_00355/miR-15a-5p axis may be one of the ways that LINC_00355 regulates the development of GC.

miR-15a-5p has been reported to play a negative regulatory role in the development of several cancers, including GC [20], bladder cancer [21], and cervical cancer [22]. Specifically, miR-15a-5p expression in GC patients was significantly decreased and miR-15a-5p overexpression suppressed GC cell proliferation and tumor invasion [23]. Zare et al. [24] demonstrated that miR-15a-5p downregulation was associated with advanced tumor grading and metastasis. miRNA is considered to regulate gene expression by binding to the 3′-untranslated region (3′-UTR) of the target genes and thus has a wide range of biological functions [25–27]. For instance, miR-15a-5p deletion suppressed the GC cell proliferation, monolayer colony formation, invasion and migration, and xenograft formation in vivo by targeting yes-associated protein 1 [20]. Here, we confirmed that PHF19 is one of the downstream targets of miR-15a-5p. PHF19 is a member of polycomblike proteins and can regulate the enzymatic activity and recruitment of polycomb inhibition complex 2 (PRC2) [28, 29]. The dysregulation of PHF19 expression is associated with the development of several cancers, including GC [30]. Previous studies have found a positive correlation between PHF19 expression and glioblastoma progression [31]. PHF19 silencing reduced the proliferation, and induced apoptosis and cell cycle stagnation in ovarian cancer cells [32]. Moreover, the overexpression of PHF19 was associated with the paclitaxel resistance of GC patients [33]. Additionally, a recent study have indicated that the downregulation of PHF19 could suppress the proliferation and migration of GC cells [30]. The findings highlight the positive regulatory role of PHF19 in cancer development. The present study also confirmed the promotion effects of PHF19 on GC progression. However, it is worth noting that a miRNA has multiple targets. This property enables miRNAs to have multiple functions through targeting different genes and suggests that miRNAs may be involved in the development of diseases, including cancer, through a variety of regulatory directions. This feature also highlights the important role of miRNAs in the pathological process. In present study, miR-15a-5p/PHF19 axis may be one of the ways that miR-15a-5p regulates the development of GC. Furthermore, considering the targeting relationship between LINC_00355 and miR-15a-5p, and miR-15a-5p and PHF19, we demonstrated that LINC_00355 may upregulate the expression of PHF19 in GC through sponging miR-15a-5p. In addition, PHF19 overexpression restored the effect of LINC_00355 knockdown on GC progression, indicating that LINC_00355 may participate in the development of GC by upregulating PHF19 expression through sponging miR-15a-5p. Our findings suggest that LINC_00355/miR-15a-5p/PHF19 axis may be one of the ways that LINC_00355 regulates the development of GC.

## Conclusions

The present study demonstrated that LINC_00355 was highly expressed in GC tissues and cells and promoted gastric cancer progression. This promotion was achieved by sponging miR-15a-5p to regulate PHF19 expression. Our findings may provide an important clinical basis for reversing the malignant phenotype of GC.

## Supplementary Information

**Additional file 1: Supplementary Figure 1.** (a) After HGC-27 and MKN45 cells were transfected with pcDNA3.1-PHF19 plasmid or its control for 24 h, relative PHF19 mRNA expression levels were detected by quantitative real-time PCR. After HGC-27 cells were transfected with pcDNA3.1-LINC_00355 or empty vector for 24 h, the transfection efficiency was detected by quantitative real-time PCR (b), the viability of HGC-27 cells was measured by CCK-8 (c), cell migration was assessed by wound-healing assay (d), cell invasion was measured by Transwell assay (e), relative protein levels of cyclin D1, MMP9, and MMP2 were detected by Western blot (f). Correlation analysis of the expression levels of LINC_00355 and PHF19 in gastric cancer tissues (g). All values were expressed as mean ± standard deviation. *n* = 3.

**Additional file 2: Supplementary Figure 2.** Relative luciferase activity were measured using Dual-luciferase reporter assay in 293 T cells co-transfected with LINC_00355 reporter plasmid or PHF19 3′-UTR-wt/mut plasmid (12.5 pmol/well) and the candidate miRNA. All values were expressed as mean ± standard deviation. *n* = 3.

**Additional file 3: Supplementary Figure 3.** Original data of western blot.

## Data Availability

The data supporting the conclusions of this article are included in the article (SF. 3).

## Abbreviations

GC: Gastric cancer
PHF19: PHD finger protein 19
MMP9: Matrix metalloproteinase 9
LncRNA: Long noncoding RNA
ceRNA: Competitive endogenous RNA
miRNA: microRNA
cDNA: Complementary DNA
PI: Propidium iodide
PARP: Poly (ADP-ribose) polymerase
Wt: Wild type
Mut: Mutant
3′-UTR: 3′-untranslated region
