# Supplementary figures and images for: LINC_00355 promotes gastric cancer progression by upregulating PHF19 expression through sponging miR-15a-5p
Source: BMC Cancer. 2021 Jun 2;21:657. doi: 10.1186/s12885-021-08227-3 (PMC8170819; doi:10.1186/s12885-021-08227-3)

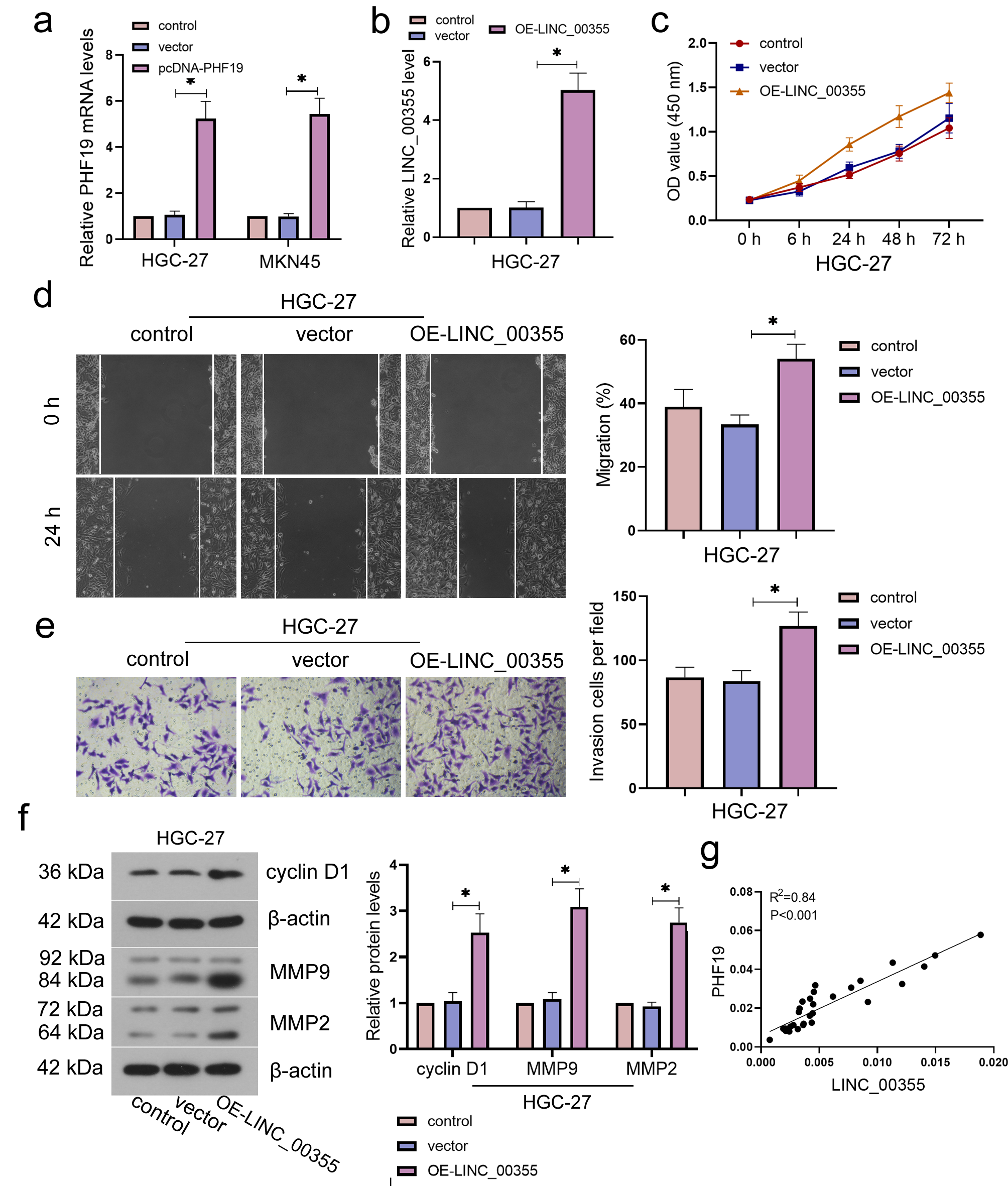

Supplement: Supplementary file 1 — Additional file 1: Supplementary Figure 1. (a) After HGC-27 and MKN45 cells were transfected with pcDNA3.1-PHF19 plasmid or its control for 24 h, relative PHF19 mRNA expression levels were detected by quantitative real-time PCR. After HGC-27 cells were transfected with pcDNA3.1-LINC_00355 or empty vector for 24 h, the transfection efficiency was detected by quantitative real-time PCR (b), the viability of HGC-27 cells was measured by CCK-8 (c), cell migration was assessed by wound-healing assay (d), cell invasion was measured by Transwell assay (e), relative protein levels of cyclin D1, MMP9, and MMP2 were detected by Western blot (f). Correlation analysis of the expression levels of LINC_00355 and PHF19 in gastric cancer tissues (g). All values were expressed as mean ± standard deviation. n = 3. [file 12885_2021_8227_MOESM1_ESM.tif]

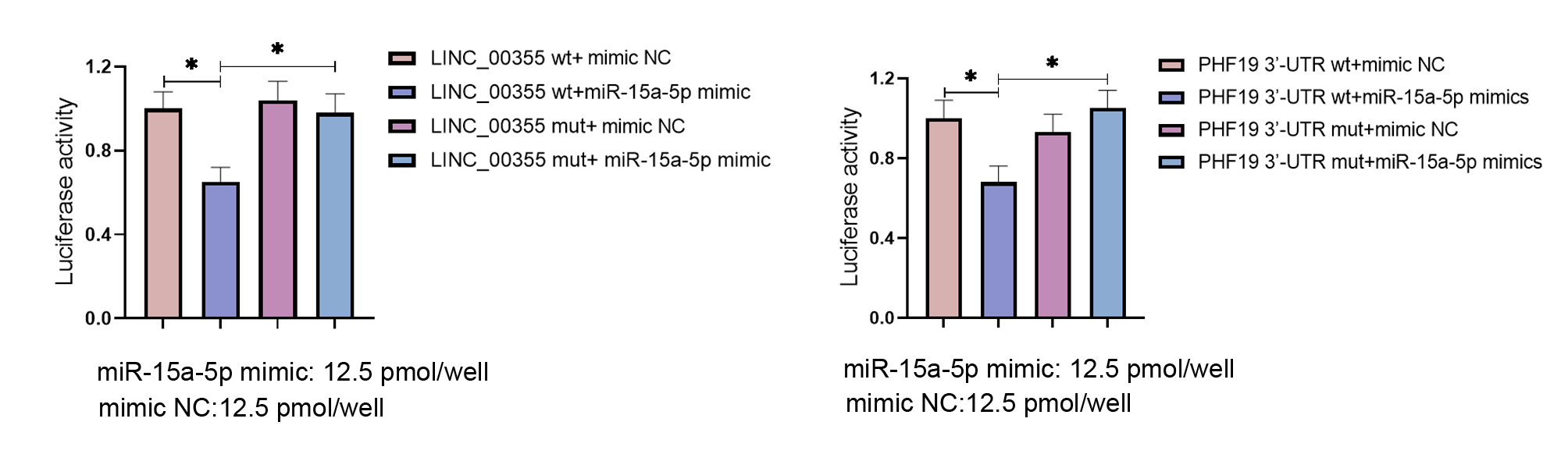

Supplement: Supplementary file 2 — Additional file 2: Supplementary Figure 2. Relative luciferase activity were measured using Dual-luciferase reporter assay in 293 T cells co-transfected with LINC_00355 reporter plasmid or PHF19 3′-UTR-wt/mut plasmid (12.5 pmol/well) and the candidate miRNA. All values were expressed as mean ± standard deviation. n = 3. [file 12885_2021_8227_MOESM2_ESM.tif]
