# Supplementary material for: LINC_00355 promotes gastric cancer progression by upregulating PHF19 expression through sponging miR-15a-5p
Source: BMC Cancer. 2021 Jun 2;21:657. doi: 10.1186/s12885-021-08227-3 (PMC8170819; doi:10.1186/s12885-021-08227-3)
Supplement: Supplementary file 3 — Additional file 3: Supplementary Figure 3. Original data of western blot. [file 12885_2021_8227_MOESM3_ESM.pdf]

Figure 2E

Groups (from left to right): control, si-NC, si-LINC_00355-1, si-LINC_00355-2

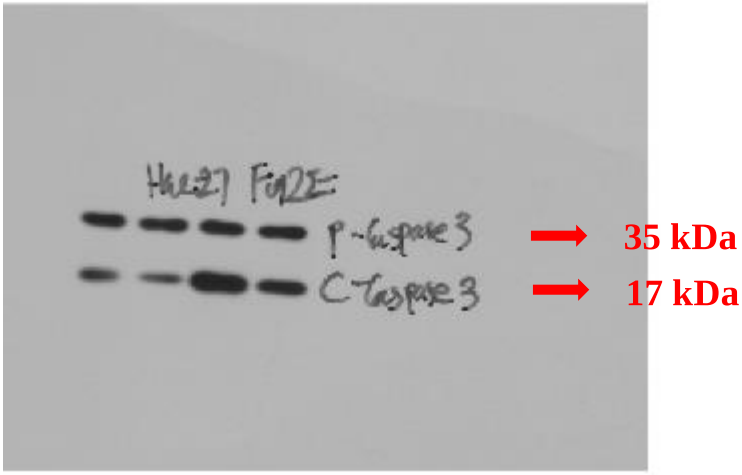

Caspase 3 (HGC-27)

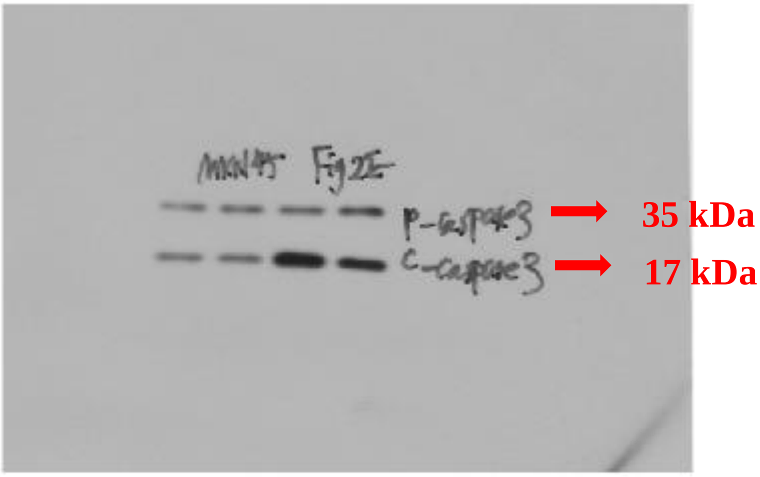

Caspase 3 (MKN45)

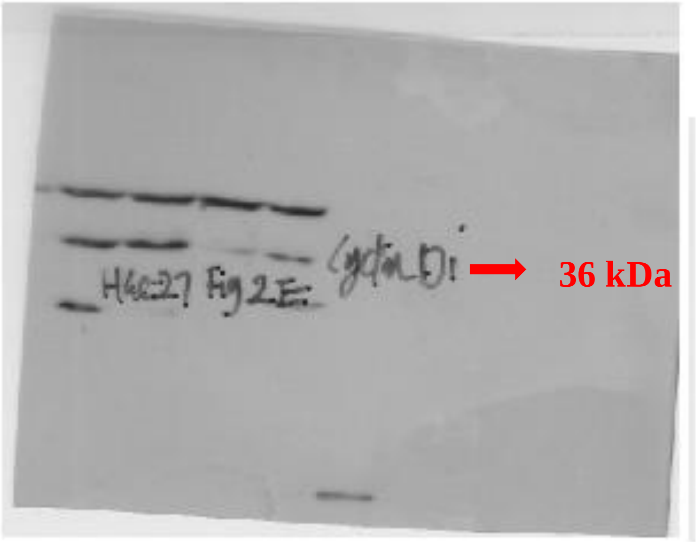

Cyclin D1 (HGC-27)

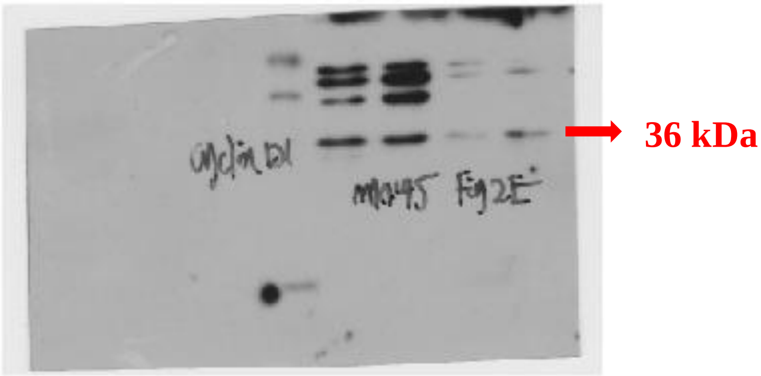

Cyclin D1 (MKN45)

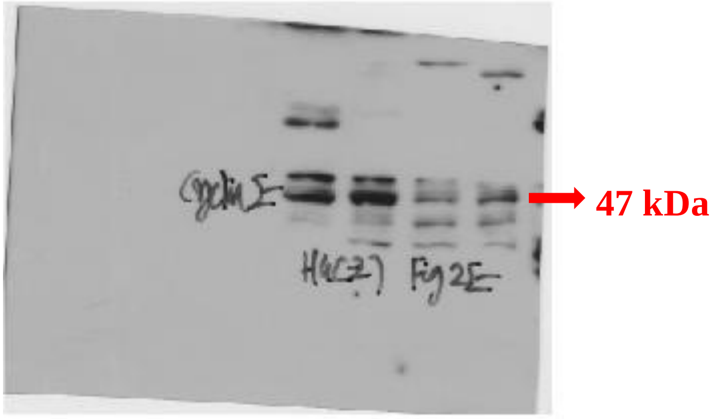

Cyclin E (HGC-27)

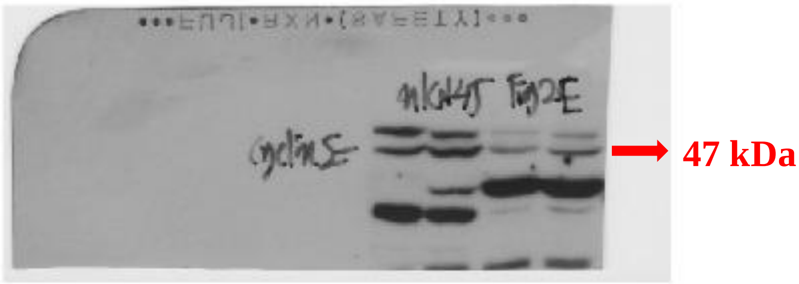

Cyclin E (MKN45)

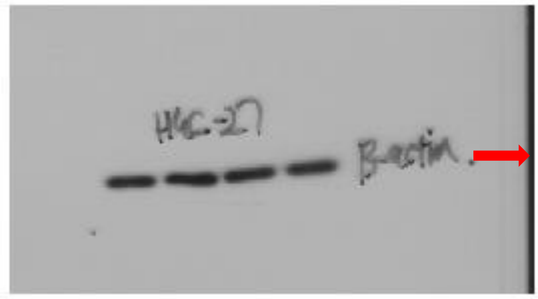

42 kDa

β-actin (HGC-27)

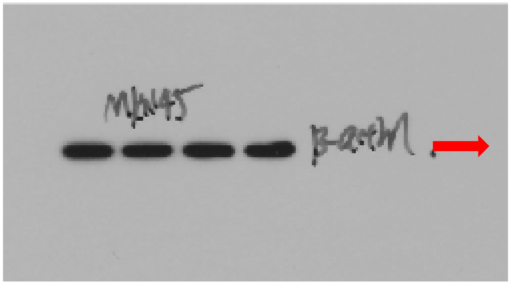

42 kDa

β-actin (MKN45)

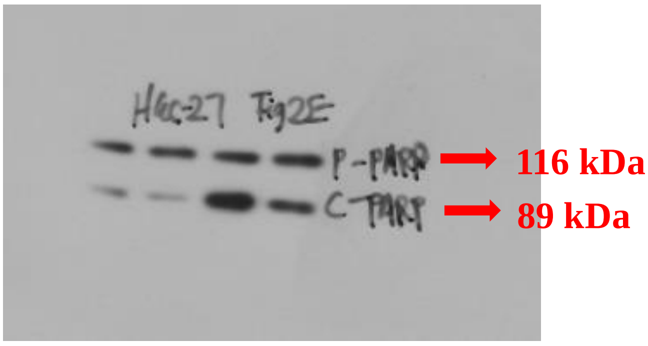

116 kDa

89 kDa

PARP (HGC-27)

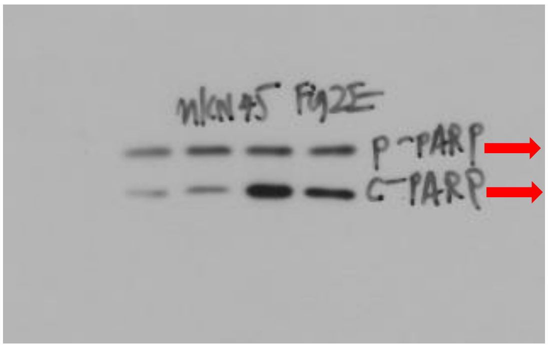

116 kDa

89 kDa

PARP (MKN45)

Figure 3C

Groups (from left to right): control, si-NC, si-LINC_00355-1, si-LINC_00355-2

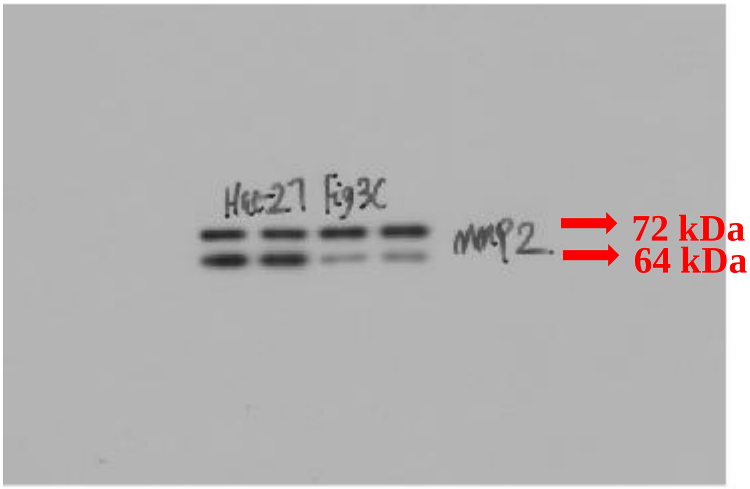

MMP2 (HGC-27)

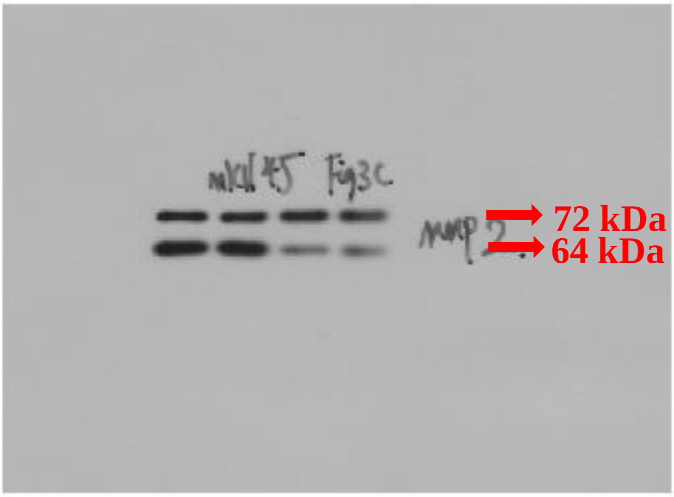

MMP2 (MKN45)

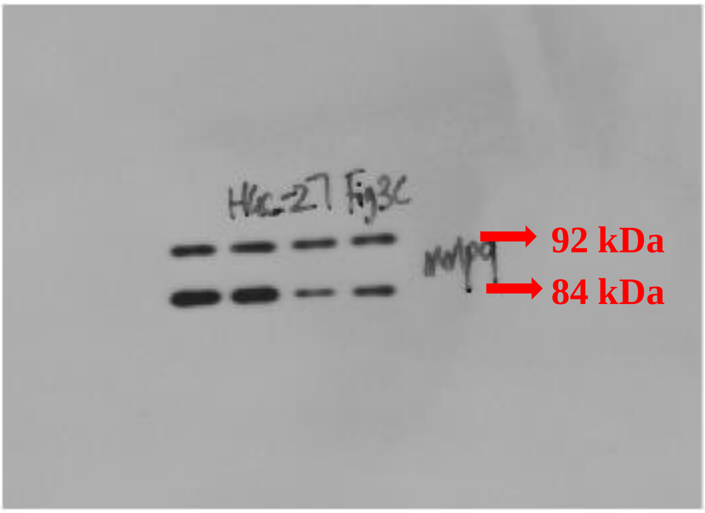

MMP9 (HGC-27)

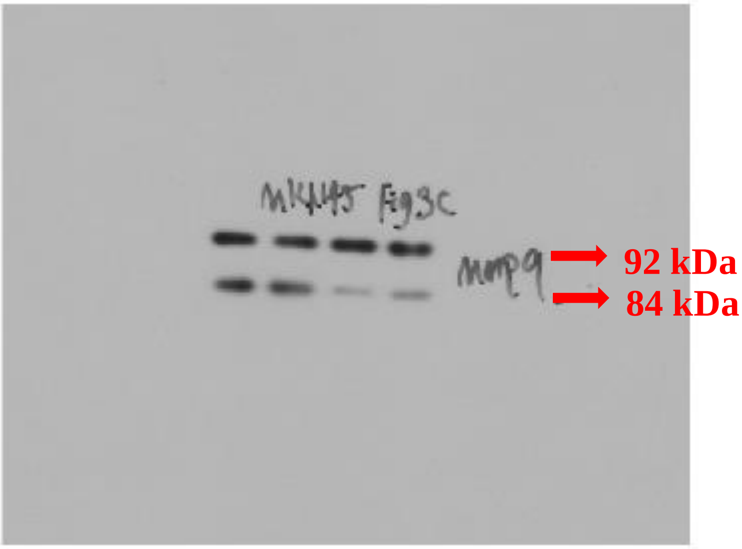

MMP9 (MKN45)

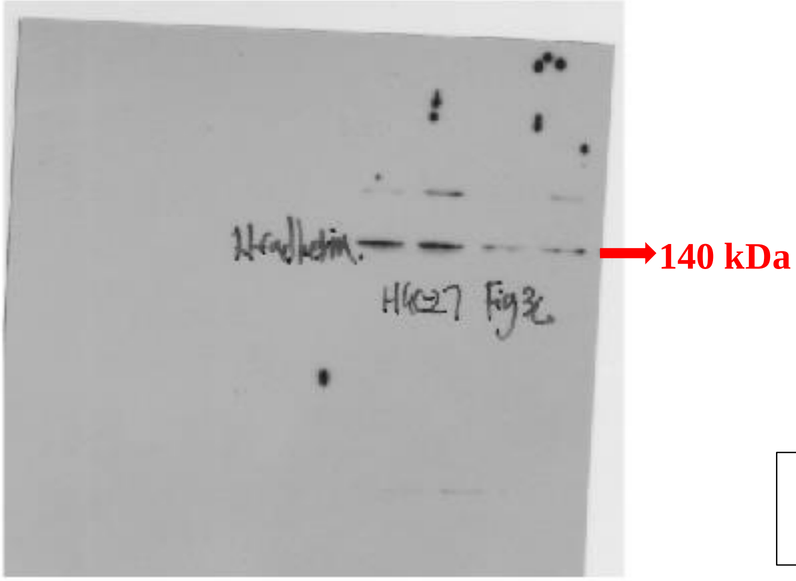

N-cadherin (HGC-27)

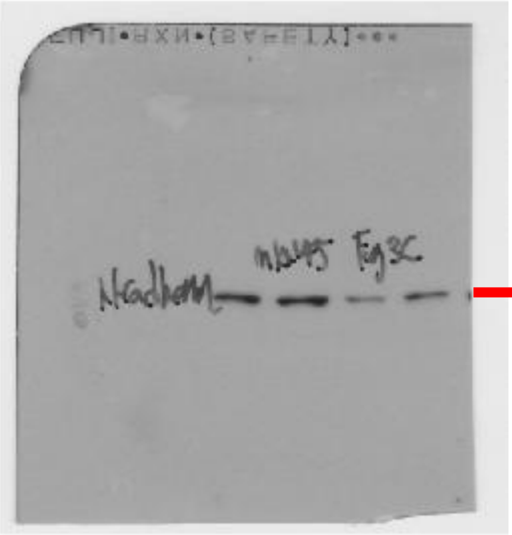

140 kDa

N-cadherin (MKN45)

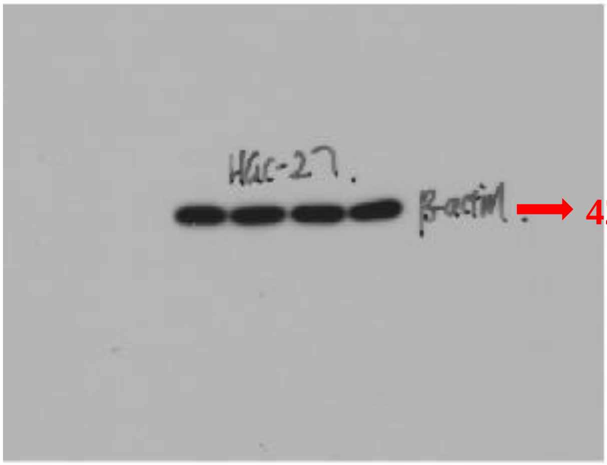

42 kDa

β-actin (HGC-27)

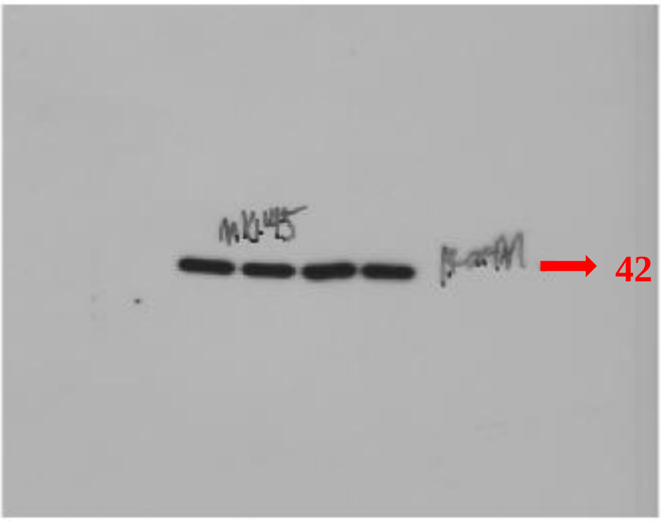

42 kDa

β-actin (MKN45)

Figure 4C

Groups (from left to right): control, si-NC, si-LINC_00355-1, si-LINC_00355-2

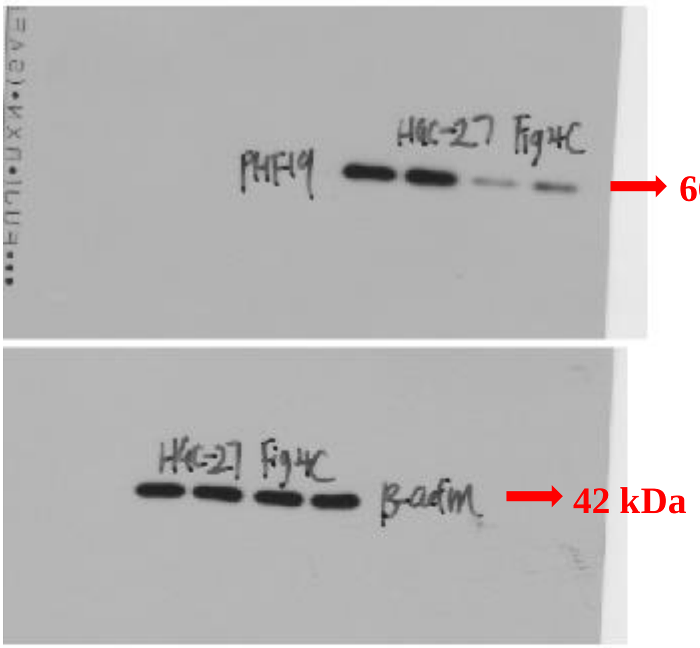

66 kDa

PHF19 (HGC-27)

42 kDa

β-actin (HGC-27)

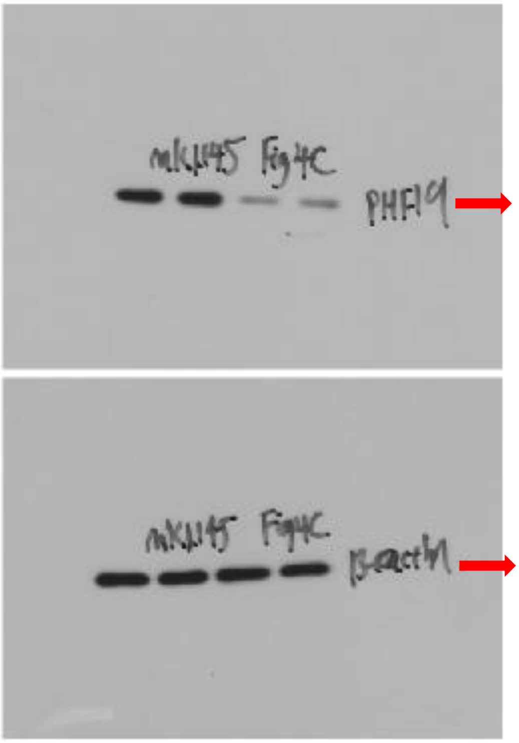

66 kDa

PHF19 (MKN45)

42 kDa

β-actin (MKN45)

Figure 4F

Groups (from left to right): control, mimic NC, miR-15a-5p mimic, inhibitor NC, miR-15a-5p inhibitor

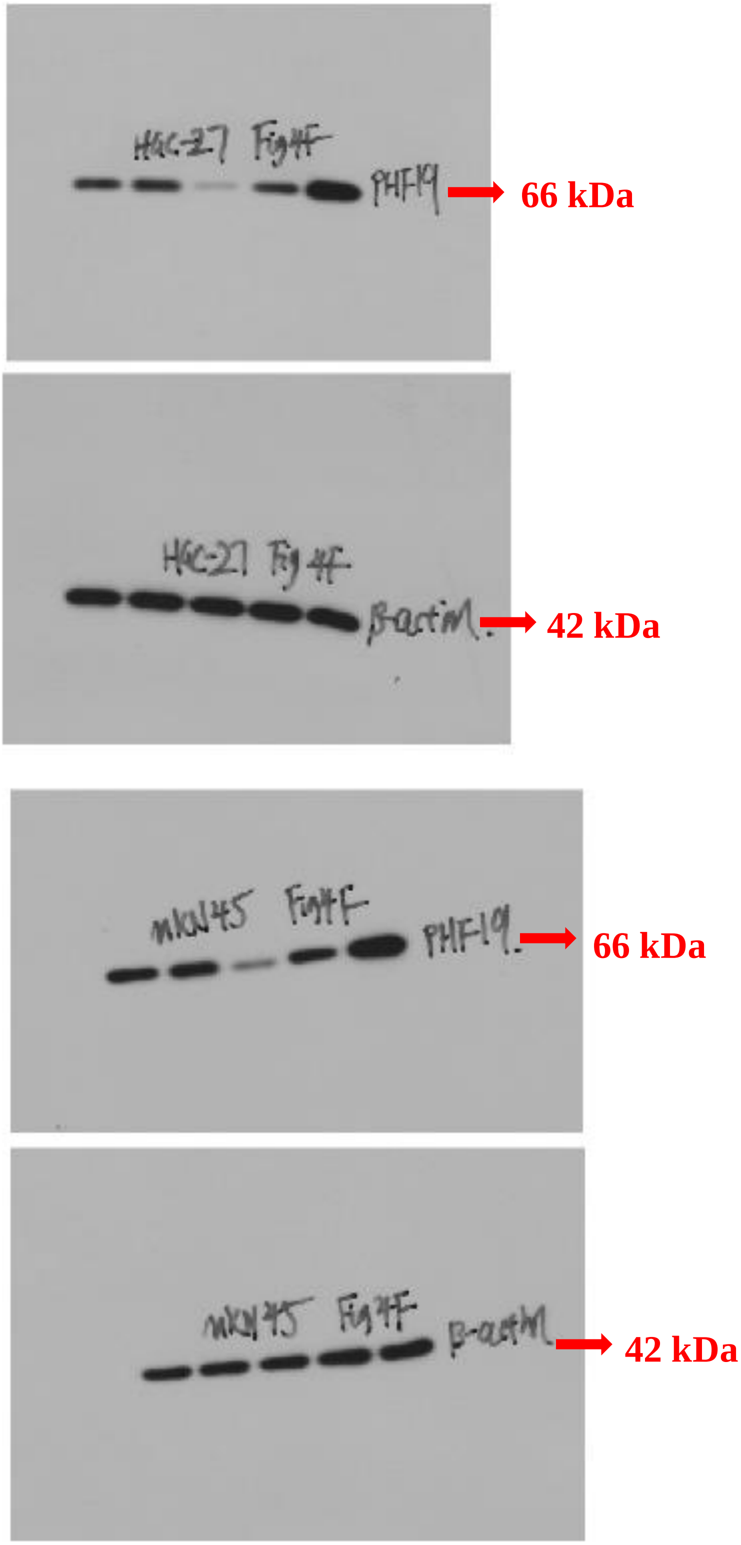

PHF19 (HGC-27)

β-actin (HGC-27)

PHF19 (MKN45)

β-actin (MKN45)

Figure 5E

Groups (from left to right): si-NC+vector, si-NC+pcDNA-PHF19, si-LINC_00355+vector, si-LINC_00355+pcDNA-PHF19

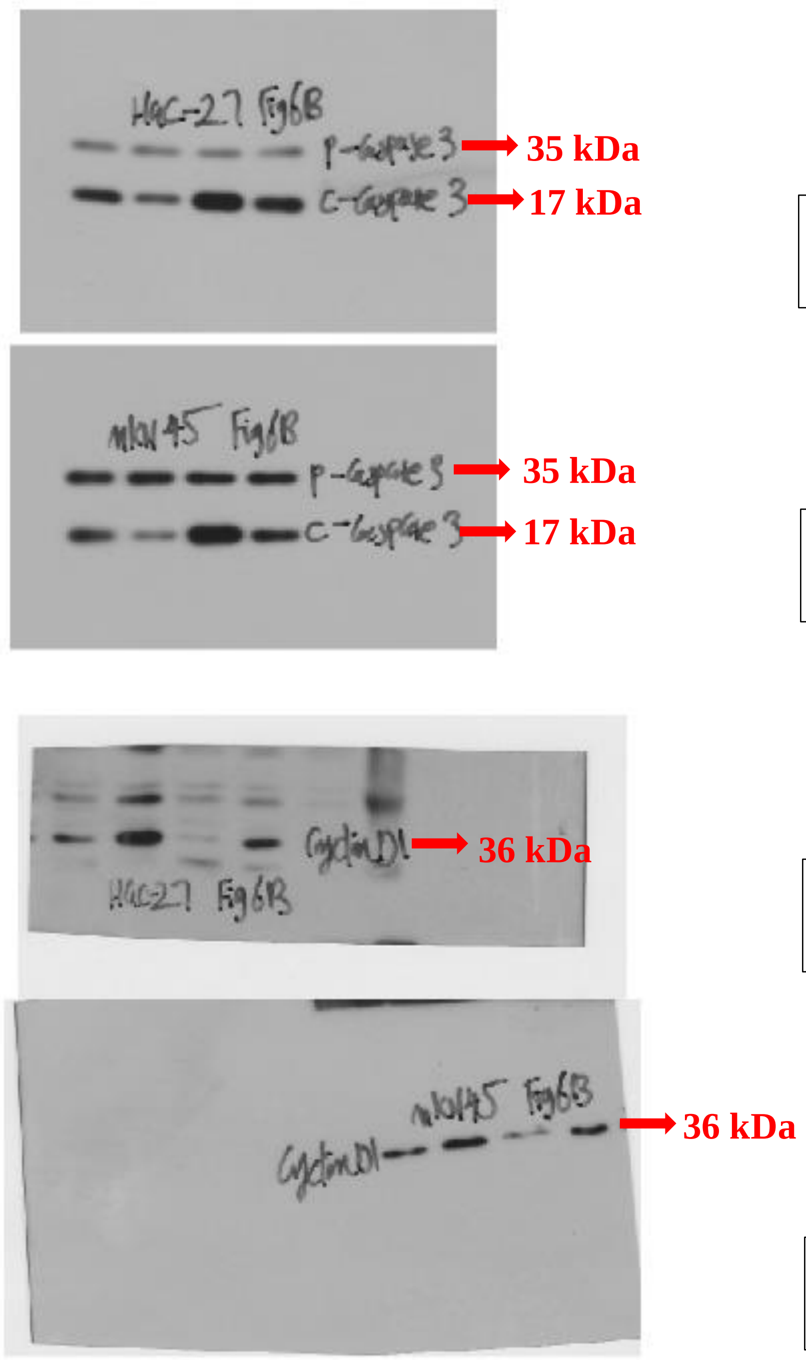

Caspase 3 (HGC-27)

Caspase 3 (MKN45)

cyclinD1 (HGC-27)

cyclinD1 (MKN45)

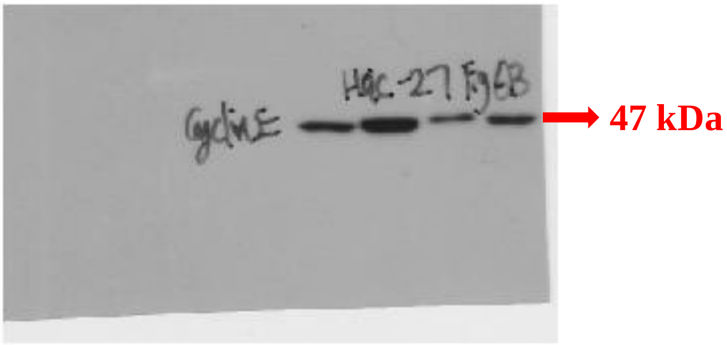

Cyclin E (HGC-27)

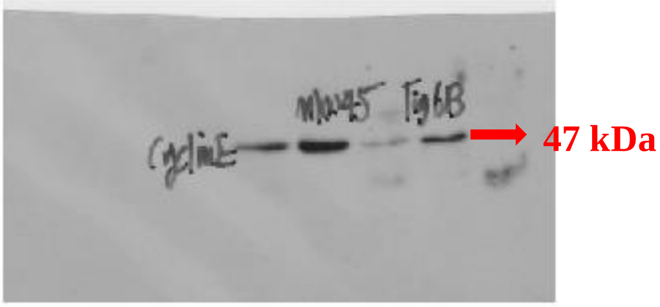

Cyclin E (MKN45)

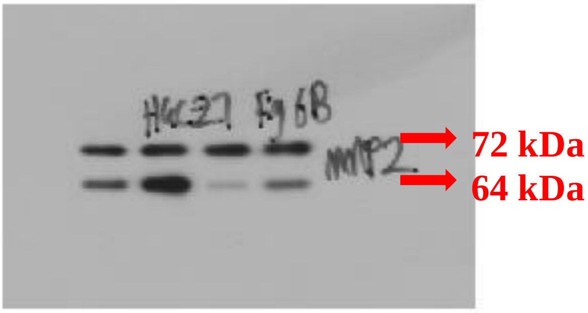

MMP2 (HGC-27)

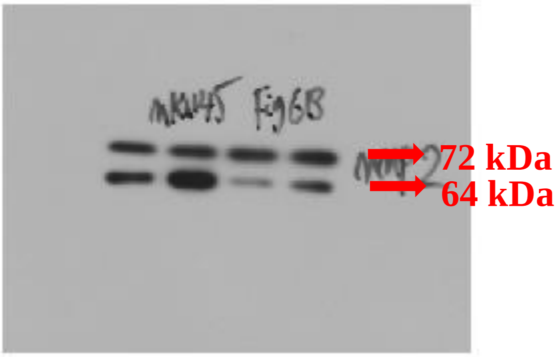

MMP2 (MKN45)

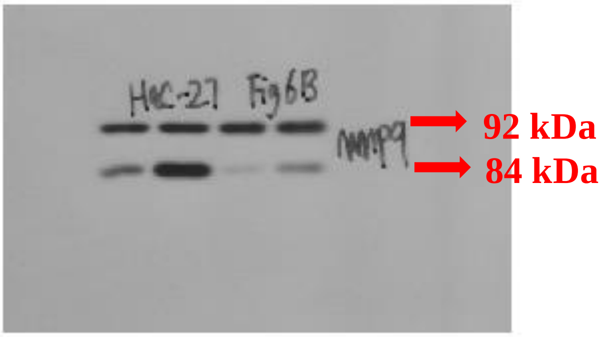

MMP9 (HGC-27)

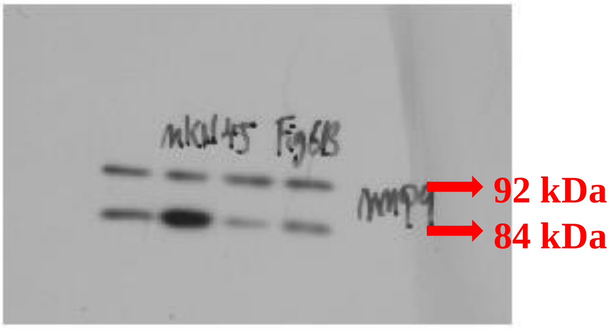

MMP9 (MKN45)

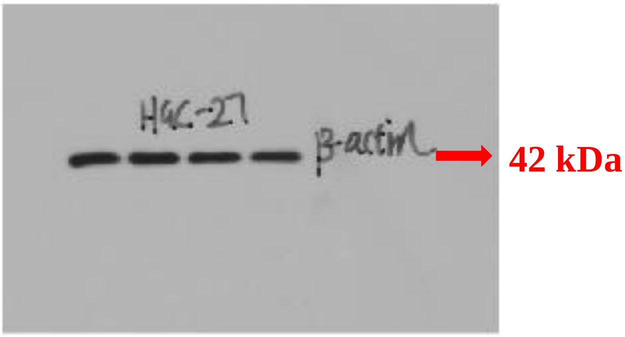

β-actin (HGC-27)

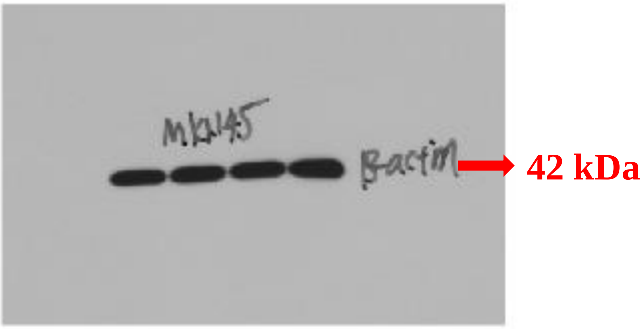

β-actin (HGC-27)
